# Job Satisfaction Among Plastic Surgery Residents in Canada

**DOI:** 10.1177/22925503211007237

**Published:** 2021-04-27

**Authors:** Andrea E. Copeland, Victoria Mackinnon, Daniel E. Axelrod, Forough Farrokhyar, Ronen Avram, Christopher J. Coroneos

**Affiliations:** 1 Division of Plastic Surgery, Department of Surgery, McMaster University, Hamilton, Ontario, Canada; 2 Michael G. DeGroote School of Medicine, McMaster University, Hamilton, Ontario, Canada; 3 Division of Orthopaedic Surgery, Department of Surgery, McMaster University, Hamilton, Ontario, Canada; 4 Department of Health, Evidence and Impact, McMaster University, Hamilton, Ontario, Canada

**Keywords:** accreditation, burnout, psychological, job satisfaction, internship and residency, wellness

## Abstract

**Objective:**

Resident wellness is a focus of medical training and is prioritized in both Canadian and American accreditation processes. Job satisfaction is an important component of wellness that is not examined in the literature. The purpose of this study was to analyze job satisfaction in a national sample of plastic surgery residents, and identify factors that influence satisfaction.

**Methods:**

We designed a cross-sectional survey adapted from existing instruments, with attention to thorough item generation and reduction as well as pilot and clinical sensibility testing. All plastic surgery residents at Canadian institutions were surveyed regarding overall job satisfaction as well as personal- and program-specific factors that may affect satisfaction. Predictors of satisfaction were identified using multivariable regression models.

**Results:**

The response rate was 40%. Median overall job satisfaction was 4.0 on a 5-point Likert scale. Operative experience was considered both the most important element of a training program, and the area in most need of improvement. Senior training year (*P* < .01), shorter commute time (*P* = .04), fewer duty hours (*P* = .02), fewer residents (*P* < .01), and more fellows (*P* < .01) were associated with significantly greater job satisfaction.

**Conclusions:**

This is the first study to gather cross-sectional data on job satisfaction from a national sample of plastic surgery residents. The results from this study can inform programs in making tangible changes tailored to their trainees’ needs. Moreover, our findings may be used to inform a prospectively studied targeted intervention to increase job satisfaction and resident wellness to address North American accreditation standards.

## Abstract (fr)

**Objectif:**

Le bien-être des résidents est un point central de la formation en médecine et représente une priorité dans le processus d’agrément canadien et américain. La satisfaction au travail constitue un volet important du bien-être qui n’est pas évalué dans les publications. La présente étude visait à analyser la satisfaction au travail dans un échantillon national de résidents en plasturgie et à déterminer les facteurs qui influent sur la satisfaction.

**Méthodologie:**

Les chercheurs ont conçu un sondage transversal adapté d’outils existants, en s’attardant à la production et à la réduction de points approfondis et à un test de sensibilité clinique. Tous les résidents en plasturgie des établissements canadiens ont reçu un sondage sur leur satisfaction au travail et sur les facteurs personnels et propres à leur programme, susceptibles d’influer sur leur satisfaction. Les chercheurs ont déterminé les prédicteurs de satisfaction au moyen de modèles de régression multivariables.

**Résultats:**

Le taux de réponse s’élevait à 40%. La satisfaction au travail médiane globale obtenait un résultat de 4,0 sur l’échelle de Likert de cinq points. L’expérience opératoire était considérée à la fois comme l’élément le plus important d’un programme de formation et comme le secteur qui a le plus besoin d’être amélioré. L’année de formation senior (*P* < .01), une durée de déplacement plus courte (*P* = .04), un moins grand nombre d’heures de garde (*P* = .02) et de résidents (*P* < .01) et un plus grand nombre de boursiers postdoctoraux (*P* < .01) ont été associés à une satisfaction au travail considérablement plus marquée.

**Conclusions:**

C’est la première étude pour colliger des données transversales sur la satisfaction au travail auprès d’un échantillon national de résidents en plasturgie. Les résultats de cette étude peuvent éclairer les programmes pour apporter des changements tangibles adaptés aux besoins de leurs stagiaires. De plus, nos observations pourraient éclairer une intervention prospective ciblée pour accroître la satisfaction au travail et le bien-être des résidents afin de satisfaire aux normes d’agréments nord-américaines.

## Introduction

Resident wellness has gained significant attention in recent years and has become a focus of medical education.^1,2^ “Commitment to self” (physician health and well-being) was recently added as a professionalism sub-competency in the CanMEDS framework.^3^ The Royal College also prioritizes resident wellness in the accreditation process.^4^ Similarly, in 2017, the American Council for Graduate Medical Education (ACGME) mandated the implementation of wellness initiatives as a requirement across all American residency programs.^5^

Surgical residents face unique wellness challenges including long work hours, unpredictable schedules, and the responsibility of developing technical skills.^6^ Not surprisingly, rates of burnout are consistently higher among surgical residents when compared to non-surgical residents^7-10^ and attending physicians.^6,11^ Resident wellness is not simply the absence of burnout^12^; job satisfaction, which describes how trainees feel about their situation at work,^13,14^ is equally important in determining resident wellness.^15,16^ High job satisfaction is associated with greater productivity and efficiency, reduced turnover, and improved patient safety.^1,17^ Promoting satisfaction benefits both the personal well-being of residents and also patient care.

Whereas physician burnout has been extensively studied and has a validated outcome scale (the Maslach Burnout Inventory),^18^ resident job satisfaction has been less studied and the literature lacks evidence for interventions to improve it.^14^ Moreover, interventions such as the ACGME’s 80-hour duty hour restriction have notably not improved resident wellness, despite their detriments to board certification exams results and patient care.^19^ It is clear a more nuanced understanding of resident wellness is required to address gaps in our approach to modern surgical training.

Job satisfaction has not been studied among plastic surgery residents. Previous studies suggest that resident satisfaction is unique to the specific specialty and training program.^15,20,21^ It is therefore important to study specialty- and program-specific resident satisfaction in order to provide tailored program adjustments and trainee recommendations. Our objectives were to obtain a cross-sectional view of job satisfaction among a national sample of plastic surgery residents and to identify factors, both personal- and program-specific, associated with job satisfaction. Understanding these factors will allow the development of integrated approaches to target wellness in the resident population; this would address a significant gap in the surgical literature.

## Methods

This study was reviewed by the research ethics board review at our institution. Participating residents were informed that survey results were anonymous and that results would be reported in aggregate.

### Survey Development

Development of the survey began with item generation. A review of the literature was performed by a single reviewer to identify predictors of job satisfaction among surgical residents. Variations of the following search terms were used: *resident, satisfaction, survey, surgical*. The following 12 domains were identified: operative experience, teaching opportunities, quality of teaching, research opportunities, mentorship, workplace climate, level of supervision, workload, feedback, quality of life, administrative components, and training for future independent practice. Ten demographic factors (age, gender, marital status, number of children, post-graduate year, geographic location, ethnicity, commute time, average debt, and domestic vs foreign medical graduate) and 4 program-factors (number of residents in program, number of fellows, hours worked per week, and call shifts worked per month) were also summarized as potential predictors of job satisfaction. Finally, a single question evaluating overall job satisfaction was included. While “overall satisfaction” is not specific, single-item questions about overall satisfaction are found to have strong convergent validity (*r* = 0.63) when other questions within the survey are considered components of overall satisfaction.^22^ This question was intentionally asked at the end of the survey so respondents would understand that the preceding domains were considered components of overall satisfaction. Questions that indirectly evaluated job satisfaction were adapted from a previous survey of general surgery residents.^23^

Survey questions were generated through unstructured interviews with plastic surgery faculty and item reduction was completed using the Delphi method. The survey was then piloted among 20 current orthopaedic surgery residents at the author’s institution. Feedback was elicited in a semi-structured email regarding its salience, flow, wording, interpretability, ease of administration, and time required for completion.^24,25^ Following pilot testing, clinical sensibility testing was completed by 7 recent plastic surgery graduates from the author’s institution to assess comprehensiveness, clarity, and face validity of the questionnaire, using questions from the tool published by Burns et al.^25^

### Survey Administration

The survey (see Online Appendix, Supplemental Digital Content 1) was administered in English using a web-based survey platform (SurveyMonkey). All current (September 2019) plastic surgery residents at Canadian training institutions were emailed. As this survey was intended to inform us about current resident satisfaction, a specific sample size was not targeted; instead all residents were surveyed. A cover letter was provided including survey objectives,^26^ rationale for respondent selection,^26^ department stationary,^27^ assurance of confidentiality,^27^ estimated time required,^28^ and survey deadline.^29^ Responses were anonymous. No incentive was provided. A reminder email was sent out 2 weeks later. After 1 month, the study was closed.

### Data Analysis

Summary statistics were calculated for all survey responses. All survey items were analyzed in univariate regression models with overall job satisfaction as the dependent variable. Covariates that significantly predicted overall satisfaction (*P* < .05) were carried forward into a multivariable regression model. A 10 observation per covariate rule of thumb was utilized to prevent overfitting the final model.^30^ Given 56 unique survey responses, the final multivariate model was limited to 6 covariates. Goodness of fit of the model was reported through R^2^ values. R (Open Access, Version 3.6.1) was utilized for all statistical analyses. A biostatistician was consulted to help develop the statistical analysis plan and conduct all analyses.

## Results

### Demographics of Canadian Plastic Surgery Residents

Responses were received from 56 plastic surgery residents; 40% (56/140). Fifty-two percent of respondents are male. Each post graduate year (PGY) contributes at least 13% of the total survey responses, with the largest proportion (29%) derived from the PGY3 cohort. Most respondents are between the ages of 26 to 30 (70%), and either in a relationship (45%) or married (41%). Only 9% of respondents have children. Residents most commonly commute between 5 and 20 minutes to work (77%; Table 1).

**Table 1.** Resident Demographics.

Resident Factor	n (%)
Post graduate year (PGY)	
1	8 (14.3)
2	11 (19.6)
3	16 (28.6)
4	7 (12.5)
5	14 (25.0)
Research year	
Yes	2 (3.6)
No	54 (96.4)
Age	
25 or younger	4 (7.1)
26-30	39 (69.6)
>30	13 (23.2)
Gender	
Female	25 (44.6)
Male	29 (51.8)
Prefer not to disclose	2 (3.6)
Relationship status	
Single	7 (12.5)
In a relationship	25 (44.6)
Married or common law	22 (39.3)
Prefer not to disclose	2 (3.6)
Children	
Yes	5 (8.9)
No	50 (89.3)
Prefer not to disclose	1 (1.8)
Commute (minutes)	
Less than 5	4 (7.1)
5-10	17 (30.4)
10-20	26 (46.4)
20-30	6 (10.7)
>30	3 (5.4)

### Demographics of Canadian Plastic Surgery Training Programs

The median number of residents per program is 10 (range 9-30) and fellows is 0 (range 0-20). The majority of residents work 80 to 100 hours per week (57%) and 5 to 8 call shifts per month (66%). The most demanding post-graduate years are reported as PGY2 (30%), PGY4 (29%), and PGY3 (21%; Table 2).

**Table 2.** Residency Program Factors.

Program factor	N (%)
Hours per week	
<60	4 (7.0)
60-70	6 (10.7)
70-80	9 (16.0)
80-90	17 (30.5)
90-100	15 (26.8)
>100	5 (9.0)
Call shifts per month	
4 or less	7 (12.5)
5-6	20 (35.7)
7-8	17 (30.4)
9-10	12 (21.4)
11 or more	0
Program factor	Median (range)
Number of Residents	10 (9, 30)
Number of Fellows	0 (0, 20)

### Overall Resident Job Satisfaction

The median overall satisfaction score is 4.0 (range 1-5) on a 1 (strongly disagree) to 5 (strongly agree) Likert scale. Questions regarding happiness with work and feelings of fitting in are also median 4.0. The median score for whether residents have considered leaving their training program is 1.0. When asked whether they would change their program ranking now that they have had experience in their program, 79% indicate it would remain the same.

### Satisfaction With Domains of Training

Median satisfaction is 4.0 out of 5 for all domains except workload (3.5) and feedback (3.0; Table 3). When asked to rank the 5 most important elements of a training program, operative experience is most commonly ranked first (50%), followed by ability of program to prepare resident for next phase of career (29%), and collegial relationships within the program (11%; Table 4). Subgroup analysis shows that this first, second, and third ranking is consistent across post-graduate years and genders. Conversely, workload, research, and feedback are not ranked as the most important domain by any group, and only 7%, 13%, and 14% of residents rank them in the top 3, respectively (Table 4). When asked which domain could be improved at the resident’s training program, operative experience is chosen most frequently (n = 11, 20%). A summary of free-text responses for causes of stress in residency is found in Table 5.

**Table 3.** Resident Satisfaction With Program Domains.

Program domain	Median satisfaction (range)
Operative volume	4 (1, 5)
Operative independence	4 (1, 5)
Formal teaching	4 (1, 5)
Informal teaching	4 (1, 5)
Research opportunities	4 (1, 5)
Relationships with other residents	4 (1, 5)
Relationships with staff	4 (1, 5)
Mentorship	4 (1, 5)
Feedback given to resident	3 (1, 5)
Workload	3.5 (1, 5)
Ability of program to prepare resident for next stage of their career	4 (1, 5)

**Table 4.** Most Important Elements of a Residency Training Program.

Program domain	Ranked 1st n (%)	Ranked 2nd n (%)	Ranked 3 rd n (%)
Operative experience	28 (50.0)	20 (35.7)	7 (12.5)
Ability of program to prepare resident for next phase of their career	16 (28.5)	13 (23.2)	9 (16.0)
Collegial relationships within the program	6 (10.5)	4 (7.0)	14 (25.0)
Teaching experience	3 (5.5)	9 (16.0)	7 (12.5)
Mentorship	3 (5.5)	3 (5.5)	7 (12.5)
Feedback given to resident	0	3 (5.5)	5 (8.9)
Research opportunities	0	2 (3.6)	5 (9.0)
Workload	0	2 (3.6)	2 (3.6)

**Table 5.** Causes of Resident Stress in Residency.

Cause of stress	n (%)
Work-life balance	20 (38.5)
Managing staff expectations	8 (15.4)
Career planning	7 (13.5)
Royal College exam	5 (9.6)
Research	3 (5.8)
Support on call	2 (2.8)
Technical competence	1 (1.9)
Administrative responsibilities	1 (1.9)
Patient outcomes	1 (1.9)
Financial	1 (1.9)
Feedback	1 (1.9)
Unpredictability of schedule	1 (1.9)
Collegial relationships	1 (1.9)

### What Factors Influence Trainee Satisfaction?

Multivariable regression analysis demonstrates decreased satisfaction among residents working more hours per week (mean difference [MD]: −0.22, −0.41 to −0.04, *P* = .02), with longer commute times (MD: −0.29, −0.56 to −0.01, *P* = .04), in larger programs (MD: −0.13 for each additional resident, −0.17 to −0.05, *P* < .01), and in junior years (MD: −0.69, 95% CI −1.17, 0.2, *P* < .01). Residents in programs with more fellows have greater overall satisfaction (MD: 0.12, 0.06 to 0.19, *P* < .01). Residents working fewer call shifts per month are also more satisfied but this is not statistically significant (*P* = .09; Table 6).

**Table 6.** Multivariable Regression of Overall Resident Job Satisfaction.

Resident or program factor	Multivariable analysis coefficient (95% CI)	*P* value
post graduate year (PGY) 1-2 vs 3-5	−0.69 (−1.17, −0.20)	.006^a^
Longer commute (minutes)	−0.29 (−0.56, 0.01)	.040^a^
Number of residents in program	−0.13 (−0.17, −0.05)	.001^a^
Number of fellows in program	0.12 (0.06, 0.19)	<.001^a^
Hours worked per week	−0.22 (−0.41, 0.04)	.018^a^
Call shifts worked per month	−0.23 (−0.51, −0.51)	.087

^a^ *P* < 0.05. R^2^ = 0.410.

## Discussion

This is the first study to analyze resident job satisfaction and identify factors that impact it. We used a rigorously developed survey, and a national sample. Outcomes are expressed by domains of training, and specific factors for improvement by accreditation criteria. Overall, job satisfaction is high across all domains except “feedback,” and “workload.” Operative experience is considered both the most important element of a training program, and the area in most need of improvement. Senior training year, shorter commute time, fewer duty hours, fewer residents, and more fellows are associated with significant improvement in resident satisfaction.

Overall, plastic surgery residents in Canada report high job satisfaction. This is encouraging as several studies to date paint a negative picture of job satisfaction and burnout among surgical residents.^7,9-11,21,31-36^ The domain that residents are least satisfied with is “feedback given to residents,” with respondents commenting on the need for more frequent, formal, and mandatory feedback sessions. Previous studies have shown that enhancing the frequency and quality of resident feedback enhances both teacher and learner satisfaction.^37^ This is reflected in new North American initiatives; for example, the Canadian Royal College’s Competence By Design (CBD)^38^ and ACGME’s Milestones 2.0,^39^ which both mandate frequent and structured feedback. This domain will be important to reassess after a complete CBD cohort completes residency, given that previous research shows it is responsive to change.^37^

Our data suggest that the time burden of residency is an important theme that reduces job satisfaction. For example, more duty hours per week and longer commute times are associated with lower satisfaction. In plastic surgery, a longer commute is particularly important given the majority of call is taken from home, not within the hospital. Additionally, “workload” has the second lowest satisfaction score of all program domains. Finally, when asked about causes of stress, answers relating to workload and time burden are most common. The time burden of residency training is a frequently reported issue in the literature, particularly among surgical residents.^40^ Time burden is associated with negative outcomes such as burnout and worsened mental health.^1,10,11,33,34,41^ To help mitigate these issues, national duty hour restrictions have been implemented in Canada, mandating a maximum of 70 hours per week on average and up to 100 hours per week during peak periods.^42^ Similarly, the ACGME mandates a maximum of 80 hours per week averaged over 4 weeks.^43^ In our study, 80 to 100 hours per week is the most commonly reported duty hours range; this is in contrast to the Royal College and ACGME and mandates. Residents working 60 to 80 hours per week are the most satisfied, while working more than 80 hours per week was associated with lower satisfaction. Interestingly, residents working 40 to 60 hours per week were also less satisfied; it is possible that too few hours leads to fewer opportunities for operative exposure^19^ and lower overall satisfaction. Although the literature reports no change in well-being with duty hour restrictions,^19^ logged resident hours are often under-reported.^44^ In contrast, the anonymous responses in our study are likely more accurate. Accordingly, there may represent a benefit to keeping duty hours below a certain threshold. Previous studies^15,19^ have identified the need to increase the number of allied health professionals such as nurse practitioners, physician assistants, and cast technicians to alleviate surgical residents of non-physician-oriented tasks and increase the education yield of hours spent in hospital. This allows residents to focus their time in the operating room, thereby improving wellness and job satisfaction.^19^

Although there is no literature assessing the relationship between surgical resident seniority and job satisfaction, our findings are consistent with previous studies that suggest surgical independence and autonomy predict satisfaction.^45-47^ Plastic surgery residents complete most off-service rotations in their junior years, disrupting plastic specific learning and reducing operative volume. Moreover, the hierarchical senior-junior program structure limits junior residents’ operative experience. Similarly, having more residents in a program is associated with lower job satisfaction—possibly due to more competition for operative time. Lastly, only 3 (30%) Canadian plastic surgery residency programs have more than 2 fellows across all training sites. Accordingly, our finding that more fellows is associated with increased job satisfaction may be spurious. However, it is possible that more fellows contribute to resident learning through intra-operative teaching without negatively impacting operative experience.

Operative experience is consistently considered the most important aspect of a plastic surgery training program, and is also identified as most in need of improvement. Residents comment on the need for greater operative volume and independence. Over the last few decades, there has been a reduction in resident operative volume.^48^ This was exacerbated by implementation of duty hour restrictions.^19,48,49^ Moreover, previous studies have shown that operative experience predicts job satisfaction.^45,46,50^ While our study does not show significant correlation between satisfaction with operative experience and overall job satisfaction, the importance respondents placed on operative experience is consistent with previous literature and is an appropriate reflection of the primary goals of any surgical program—to produce competent and technically proficient residents.

Finally, collegiality among medical colleagues has previously been shown to improve individual development and overall job satisfaction.^15,51^ In our study, relationships with other residents and staff is found to be the third most important element of a training program. Satisfaction with this aspect of training is high, as is satisfaction with mentorship. Residents also report feeling that they “fit in” with their program. A study surveying American general surgery residents^51^ found that residents who socialized with their attendings and those that felt they were able to turn to their attendings for support had higher job satisfaction. Similar associations have been found with both mentorship^46,52^ and workplace climate^15^ predicting job satisfaction. In Canada, the plastic surgery community is small, which likely contributes to these findings. Given that respondents indicate that collegial relationships are an important aspect of training, it likely contributes to overall job satisfaction as well.

The results from this study can be used to inform a targeted intervention to increase resident job satisfaction. The focus should be on improving those factors that correlate with high satisfaction, as well as targeting the program domains that residents consider the most important. Duty hours can be targeted at 60 to 80 hours per week and the education to service ratio can be maximized by removing unnecessary tasks and streamlining other processes so as not to negatively impact operative time. Incoming residents should be encouraged to choose housing that will minimize their commute times. Scheduled social “wellness” activities may increase program cohesiveness and improve relationships among residents and between residents and attendings. While post-graduate year and number of residents and fellows are less modifiable factors, efforts can be made to improve junior residents’ sense of autonomy, increase the number of on-service junior rotations, and improve the operative experience for all residents. For example, the preoperative briefing, intra-operative coaching, and post-operative debriefing model^53^ has been shown to improve resident autonomy and satisfaction with operative experience.^45,53^

An intervention that incorporates these factors should be formally evaluated with either qualitative improvement methodology or through prospective scientific study. Our findings on the current state of resident job satisfaction provide the baseline “pre-implementation” data that would be necessary when measuring the impact of future interventions.

There are several limitations to this study. This topic has not been previously been studied in the plastic surgery population. To address this, we designed a rigorous survey adapted from those of other specialties, with attention to thorough item generation and pilot/clinical sensibility testing. Further, all survey analyses are subject to response bias since respondents may have stronger opinions on the topic than non-respondents. Unfortunately, we were unable to perform non-responder analysis with our study design to confirm our assumptions. Our survey had a strong response rate, meeting or exceeding similar studies. Finally, the sensitive nature of the topics explored could have led to a bias toward socially desirable responses and underreporting of negative opinions. We attempted to eliminate this with assurance of confidentiality. However, given the small Canadian plastic surgery community, it is possible residents did not feel that their answers would be anonymous.

This survey was conducted before the COVID-19 pandemic. Surgical training has been disrupted due to postponement of elective surgeries and, in some cases, redeployment of residents to critical care.^54,55^ This, in addition to stresses related to safety and exposure in the workplace,^54^ has likely affected resident job satisfaction. In order to reflect these additional stressors, we will be repeating this survey in the coming months.

## Conclusions

This is the first study to gather cross-sectional data on job satisfaction from a national sample of plastic surgery residents. We identify factors associated with high satisfaction as well as the training domains most important to residents. These findings can help programs make tangible changes tailored to their trainees’ needs, including improving duty hours, commute times, operative experience, feedback, and program cohesiveness. Future research can use the results from this study to inform the implementation of a targeted intervention which should be prospectively studied to evaluate its effectiveness in improving job satisfaction and thus resident wellness. As the Royal College and ACGME now recognize that residency programs are responsible for addressing resident wellness, development of these programs is essential.

## Supplemental Material

Supplemental Material, sj-docx-1-psg-10.1177_22925503211007237 - Job Satisfaction Among Plastic Surgery Residents in Canada: A National Survey Click here for additional data file. Supplemental Material, sj-docx-1-psg-10.1177_22925503211007237 for Job Satisfaction Among Plastic Surgery Residents in Canada: A National Survey by Andrea E. Copeland, Victoria Mackinnon, Daniel E. Axelrod, Forough Farrokhyar, Ronen Avram and Christopher J. Coroneos in Plastic Surgery
