# Supplementary figures and images for: Job Satisfaction Among Plastic Surgery Residents in Canada
Source: Plast Surg (Oakv). 2021 Apr 27;30(2):151–8. doi: 10.1177/22925503211007237 (PMC9096853; doi:10.1177/22925503211007237)

**Appendix 1. Survey**


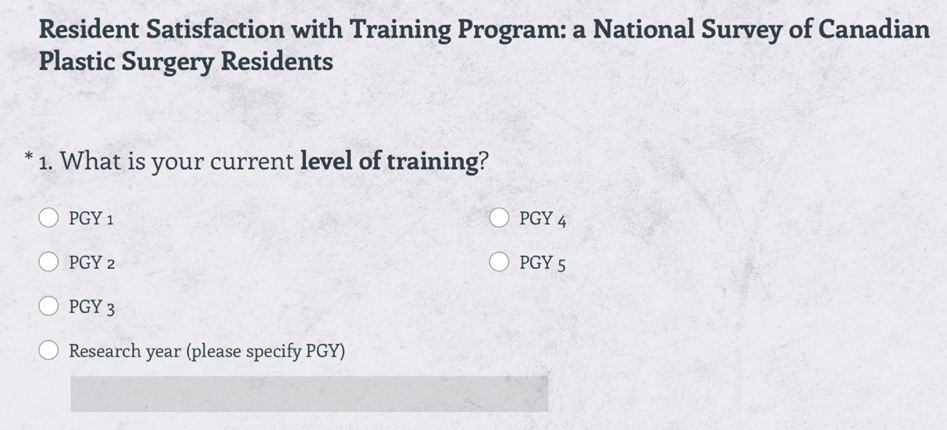


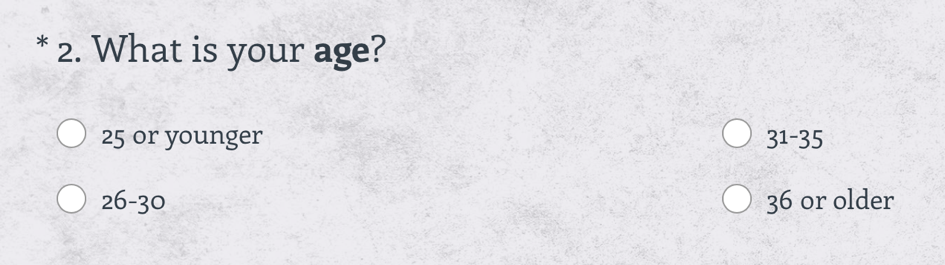


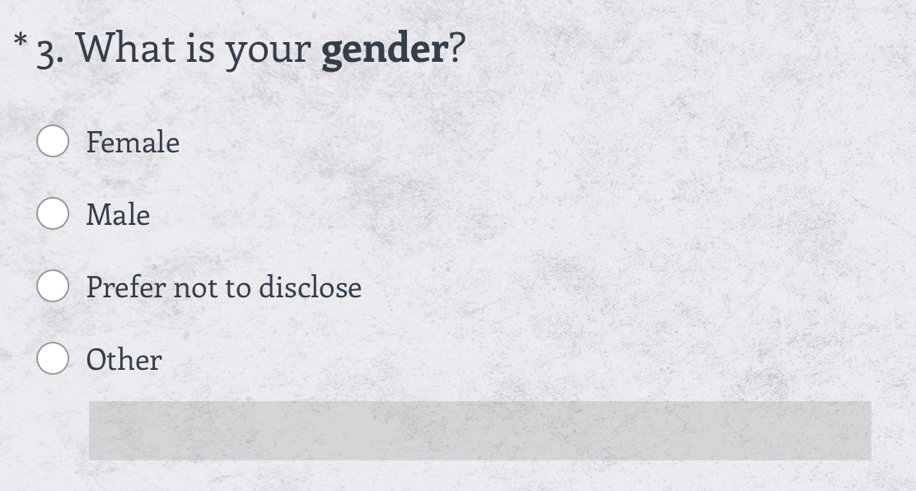


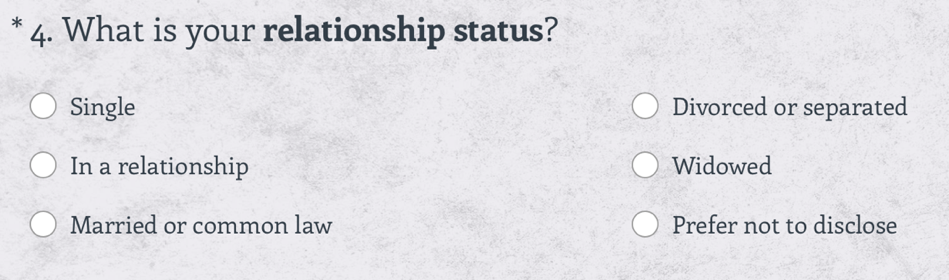


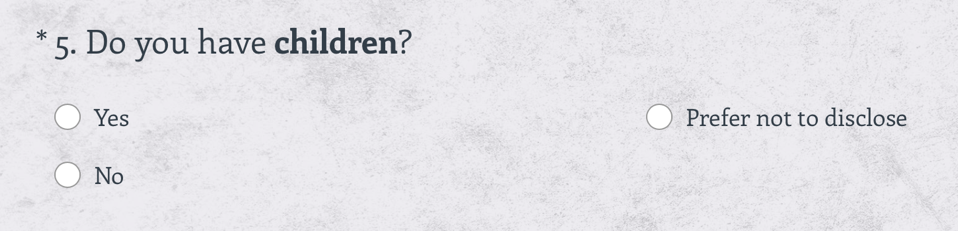


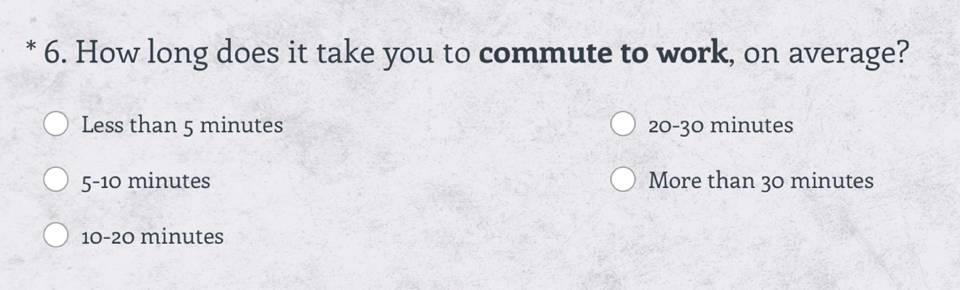


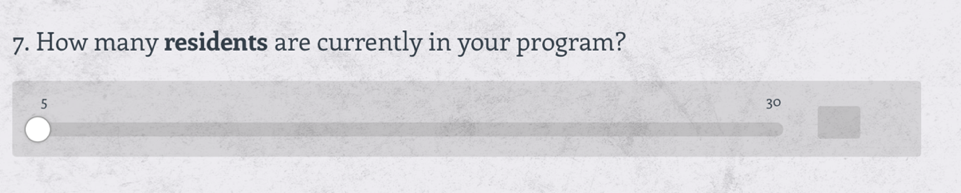


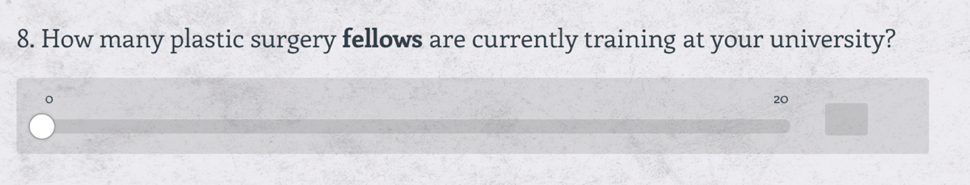


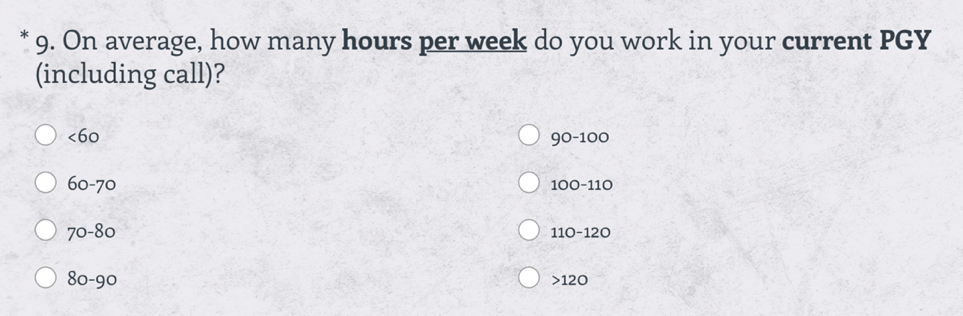


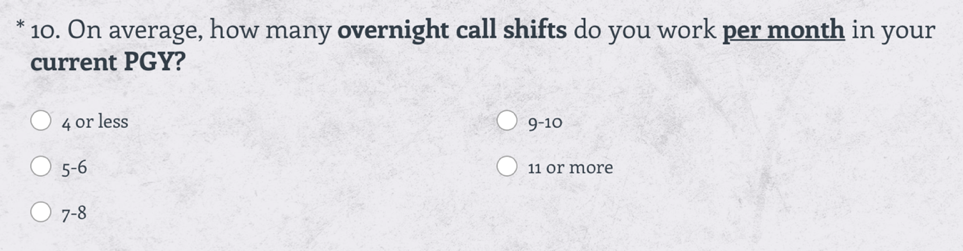


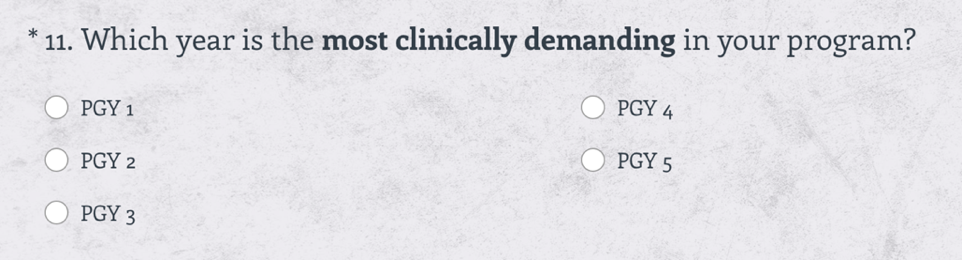


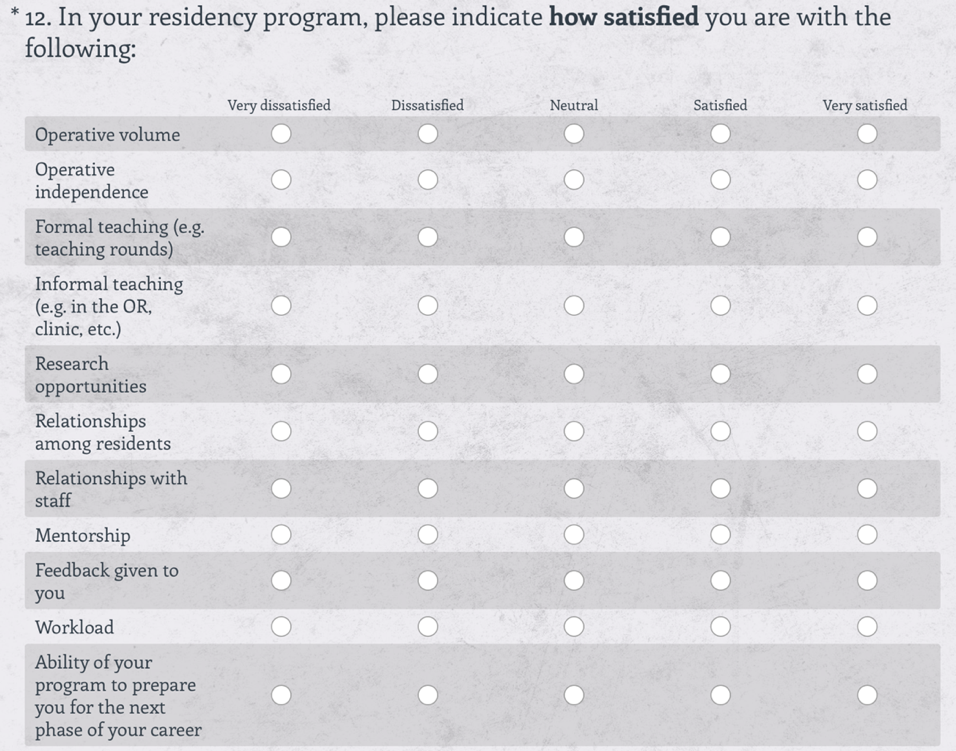


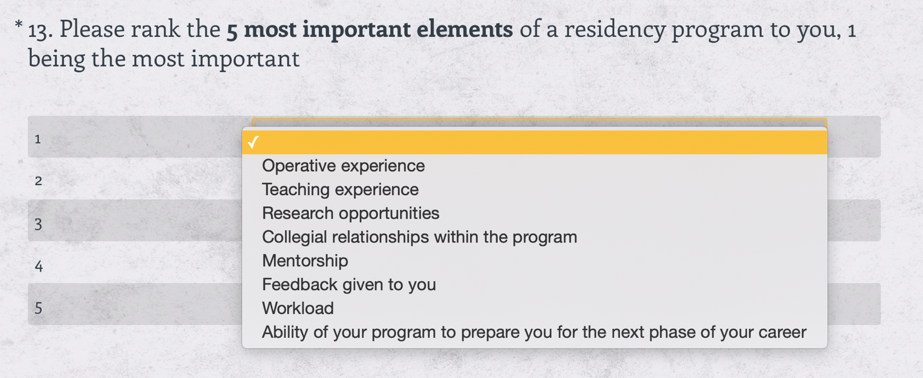


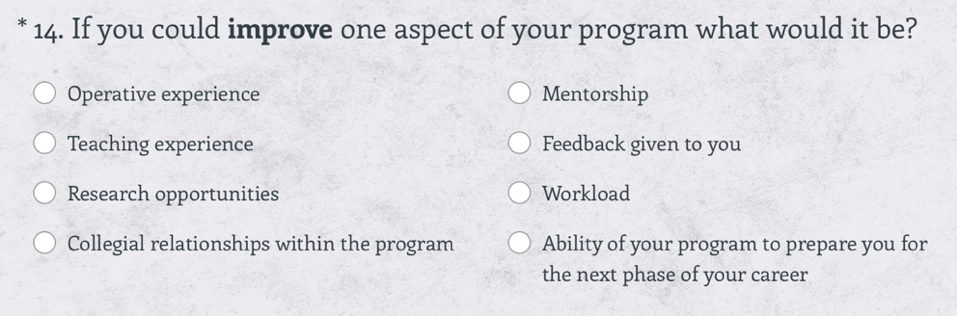


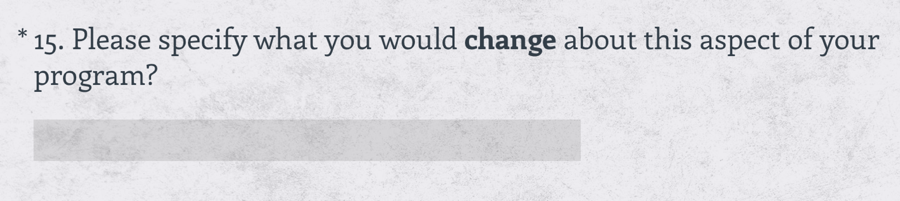


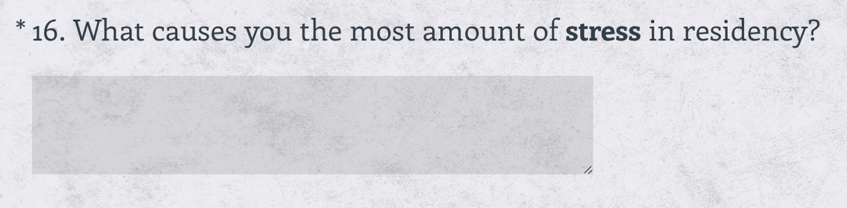


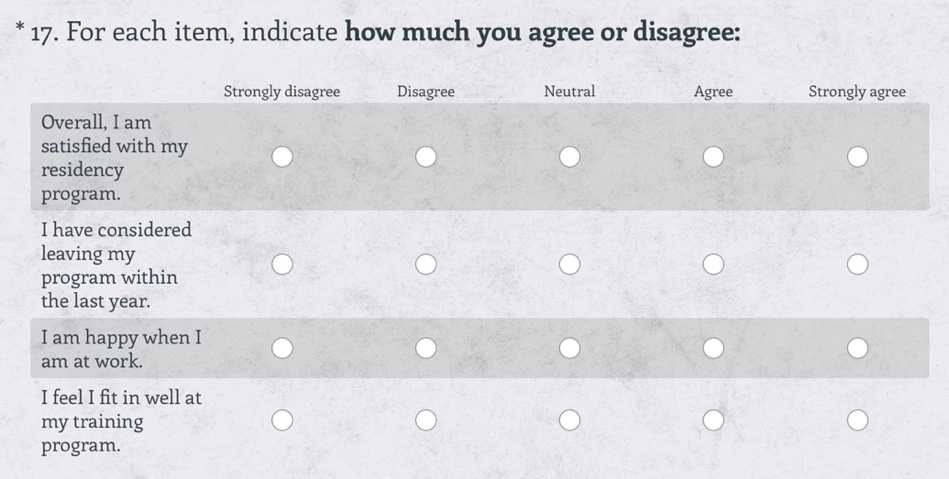


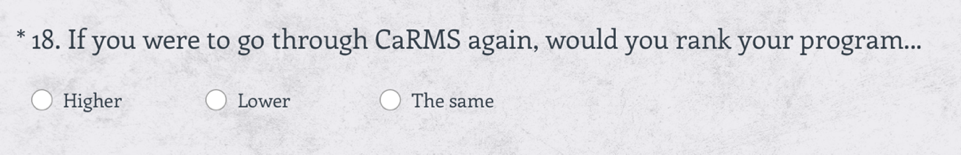


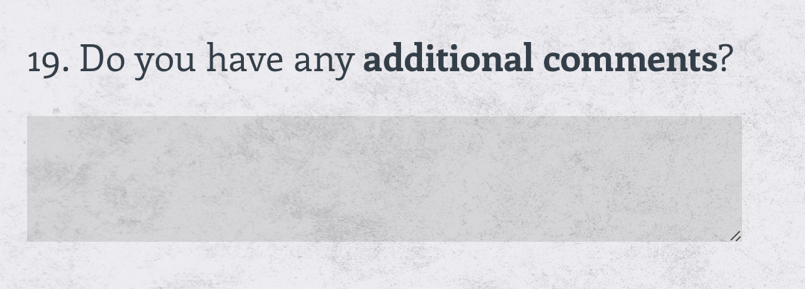

Supplement: Supplemental Material, sj-docx-1-psg-10.1177_22925503211007237 - Job Satisfaction Among Plastic Surgery Residents in Canada: A National Survey [file sj-docx-1-psg-10.1177_22925503211007237.docx]
